# Localization of the Epileptogenic Zone Using Interictal MEG and Machine Learning in a Large Cohort of Drug-Resistant Epilepsy Patients

**DOI:** 10.3389/fneur.2018.00647

**Published:** 2018-08-07

**Authors:** Ida A. Nissen, Cornelis J. Stam, Elisabeth C. W. van Straaten, Viktor Wottschel, Jaap C. Reijneveld, Johannes C. Baayen, Philip C. de Witt Hamer, Sander Idema, Demetrios N. Velis, Arjan Hillebrand

**Affiliations:** ^1^Department of Clinical Neurophysiology and MEG Center, VU University Medical Center, Amsterdam, Netherlands; ^2^Department of Radiology and Nuclear Medicine, VU University Medical Center, Amsterdam, Netherlands; ^3^Brain Tumor Center Amsterdam & Department of Neurology, VU University Medical Center, Amsterdam, Netherlands; ^4^Neurosurgical Center Amsterdam, VU University Medical Center, Amsterdam, Netherlands

**Keywords:** magnetoencephalography, presurgical evaluation, functional connectivity, refractory epilepsy, seizure freedom, beamforming

## Abstract

**Objective:** Epilepsy surgery results in seizure freedom in the majority of drug-resistant patients. To improve surgery outcome we studied whether MEG metrics combined with machine learning can improve localization of the epileptogenic zone, thereby enhancing the chance of seizure freedom.

**Methods:** Presurgical interictal MEG recordings of 94 patients (64 seizure-free >1y post-surgery) were analyzed to extract four metrics in source space: delta power, low-to-high-frequency power ratio, functional connectivity (phase lag index), and minimum spanning tree betweenness centrality. At the group level, we estimated the overlap of the resection area with the five highest values for each metric and determined whether this overlap differed between surgery outcomes. At the individual level, those metrics were used in machine learning classifiers (linear support vector machine (SVM) and random forest) to distinguish between resection and non-resection areas and between surgery outcome groups.

**Results:** The highest values, for all metrics, overlapped with the resection area in more than half of the patients, but the overlap did not differ between surgery outcome groups. The classifiers distinguished the resection areas from non-resection areas with 59.94% accuracy (95% confidence interval: 59.67–60.22%) for SVM and 60.34% (59.98–60.71%) for random forest, but could not differentiate seizure-free from not seizure-free patients [43.77% accuracy (42.08–45.45%) for SVM and 49.03% (47.25–50.82%) for random forest].

**Significance:** All four metrics localized the resection area but did not distinguish between surgery outcome groups, demonstrating that metrics derived from interictal MEG correspond to expert consensus based on several presurgical evaluation modalities, but do not yet localize the epileptogenic zone. Metrics should be improved such that they correspond to the resection area in seizure-free patients but not in patients with persistent seizures. It is important to test such localization strategies at an individual level, for example by using machine learning or individualized models, since surgery is individually tailored.

## Introduction

### Presurgical evaluation

Epilepsy surgery is a potent treatment for drug-resistant patients with a focal seizure origin. Before a patient undergoes surgery, presurgical evaluation localizes the area for resection. Magnetoencephalography (MEG) is a non-invasive technique that contributes to establish a hypothesis about the location of the epileptogenic zone (1–3), which is defined as the area that needs to be removed or disconnected to achieve seizure freedom (4). By definition, it can only be confirmed post-operatively whether the resection area corresponds to the epileptogenic zone (in case of seizure freedom) or not (in case of recurrent seizures). Epilepsy surgery attains seizure freedom in roughly half to two-thirds of patients, depending on the type of epilepsy (2, 5–7). To increase the success rate, further improvement in localizing the epileptogenic zone is needed.

### Localization of the epileptogenic zone

Several quantitative imaging metrics have been shown to localize the epileptogenic zone. Patients with epilepsy often, although not always (3, 8), show increased focal slow (delta) activity compared to controls (9), which has been used for localization of the epileptogenic zone (8, 9). Other imaging metrics have been derived from the field of connectivity and network analysis (10, 11). A functional connection is defined as the existence of statistical dependencies between time series (12). A network can be constructed from the brain regions and their connections, and its topology can be characterized using various network metrics (10).

Increased functional connectivity has been found to indicate the epileptogenic zone (13, 14) and the seizure onset zone [SOZ; the area where seizures begin (4)] (15). We previously found (16) that MEG functional connectivity was increased in the irritative zone (4). Increased functional connectivity in epileptogenic regions might augment the tendency to generate and spread seizures (14).

Regions that play a central role in networks, namely hubs (10, 17, 18), have been associated with the epileptogenic zone. Various metrics can be used to quantify the centrality of nodes (19): degree, eigenvector centrality, and betweenness centrality. Betweenness centrality has been used to identify the epileptogenic zone in both the ictal (18, 20, 21) and interictal state (17, 18, 21). The presence of hubs in or near the epileptogenic zone alludes to a role for hubs in seizure spread (10, 17, 22).

### Machine learning in epilepsy

Machine learning builds a prediction model from the data using metrics from e.g., imaging data or patient characteristics, thereby elegantly bypassing the need for multiple comparisons correction (23). The algorithm is trained using these features to classify between two or more labeled subsets. In epilepsy, such classifiers have been used to predict surgery outcome (24–26) or to identify epileptogenic regions using interictal data (27–29). Performance typically increases with larger training datasets, although classifiers have also been applied successfully to rather small clinical datasets (26, 27, 29). The trained classifiers allow for inferences at an individual level (23, 30).

### Aim and research questions

We aimed to identify metrics based on interictal MEG recordings that localize the epileptogenic zone. This paper is divided into two parts. The first part is a group level analysis to identify metrics that localize the epileptogenic zone, to address the following research questions: do the metrics overlap with the resection area? Is the overlap different in seizure-free patients compared to patients with persistent seizures? The second part is an analysis at the individual level using machine learning, investigating whether any observed group differences are relevant for individual patients. The research questions were: can the classifiers distinguish between resection and non-resection areas at the individual level? Additionally, can they distinguish between seizure-free and not seizure-free patients?

## Methods

### Patients

The patient cohort is an extension of the cohort presented in (17) and is heterogeneous regarding seizure etiology. Ninety-four patients met the following inclusion criteria: (1) They received a clinical MEG recording as part of their presurgical evaluation between 2010 and 2015 at the VU University Medical Center. (2) They subsequently underwent epilepsy surgery at the same center. (3) Surgery outcome information was available, which was assessed with the Engel classification (31) 1 year after surgery for all patients, except for three patients who had a 6 month follow up. No rules or procedures were imposed other than routine clinical care, accordingly no approval for this study by the institutional review board (Medisch Ethische Toetsingscommissie VUmc) and informed consent were needed according to the Dutch health law of February 26, 1998 (amended March 1, 2006), i.e. Wet Medisch-Wetenschappelijk Onderzoek met mensen (WMO; Medical Research Involving Human Subjects Act), division 1, section 1.2.

### MEG acquisition

Interictal MEG recordings were acquired using a whole-head system (Elekta Neuromag Oy, Helsinki, Finland) with 306 channels (102 magnetometers and 204 gradiometers). The recordings were performed inside a magnetically shielded room (Vacuumschmelze GmbH, Hanau, Germany) with the patients in supine position. Three eyes-closed resting-state recordings of typically 15 min each were recorded for clinical analysis of interictal epileptiform activity. Only one recording was analyzed in this study and chosen according to the following criteria with descending priority: (1) consisting of at least 5 min of data, (2) displaying the smallest number of artifacts, and (3) being the earlier dataset of the three recordings. The data were recorded with a sampling frequency of 1250 Hz and filtered online with a 410 Hz anti-aliasing filter and a 0.1 Hz high-pass filter. The relative position of the head to the MEG sensors was recorded continuously with 4 or 5 head-localization coils. A 3D digitizer (Fastrak, Polhemus, Colchester, VT, USA) digitized the head-localization coil positions and scalp outline (roughly 500 points). Co-registration of the scalp surface points with the patient's anatomical magnetic resonance imaging (MRI) was performed with surface-matching. Offline spatial filtering of the raw data removed artifacts using the temporal extension of Signal Space Separation (tSSS) (32) using MaxFilter software (Elekta Neuromag Oy; version 2.1), with details and parameter settings as described in (33).

### Source reconstruction

The reconstruction of neuronal sources was performed with an atlas-based beamforming approach, modified from (34). In this study, the time series of neuronal activity were reconstructed for the centroids (35) of 90 ROIs of the automated anatomical labeling (AAL) atlas (36), of which 78 were cortical ROIs (37) and 12 subcortical ROIs (excluding the cerebellar ROIs). The centroids of the MRI template were inversely transformed to the patient's co-registered MRI. Subsequently, each centroid's time series was reconstructed with a scalar beamformer (Elekta Neuromag Oy; beamformer; version 2.2.10). The beamformer acts as a spatial filter, whose weights were calculated separately for each centroid to maximally pass signals from the centroid of interest while attenuating all other signals. The weights were based on the data covariance, the noise covariance and the lead fields (calculated using a single sphere head model based on the scalp surface from the patient's anatomical MRI, and an equivalent current dipole as source model). The data covariance was based on the entire length of the selected recording in the broadband (0.5–48 Hz). The noise covariance was represented as a unity matrix. The time series (virtual electrodes) for each centroid (35) were reconstructed by projecting the broadband data through the normalized beamformer weights (38).

### Metrics

Based on the number of epochs in the shortest recording, the first 174 epochs were selected for each patient without regarding epileptiform activity or artifacts. Each epoch contained 4096 samples (3.28 s) and were analyzed in Brainwave (version 0.9.152.4.1 available from http://home.kpn.nl/stam7883/brainwave.html). Four metrics were evaluated: relative delta power, low-to-high frequency power ratio, broadband PLI, and broadband betweenness centrality.

The relative power for each time series was estimated using an offline discrete Fast Fourier Transform filter for the following frequency bands: delta (0.5–4 Hz), theta (4–8 Hz), lower alpha (8–10 Hz), upper alpha (10–13 Hz), beta (13–30 Hz), and gamma (30–48 Hz). The **relative delta power** for each ROI was used as the first metric.

The added relative power for the low frequency bands (delta and theta) were divided by the relative power for the lower alpha frequency band to obtain a low-to-high frequency power ratio. This **low-to-high frequency power ratio** for each ROI was used as the second metric.

The PLI (phase lag index) is a functional connectivity metric and measures the asymmetry in the distribution of instantaneous phase differences between two time series (39). It is robust against zero-lag phase synchronization due to volume conduction or field spread (39). For each ROI, the **broadband PLI** (0.5–48 Hz) to all other ROIs was averaged and used as the third metric.

A functional network was constructed based on the PLI values. The 90 ROIs served as nodes and the inverted PLI values (1/PLI) as edge weights. Subsequently, the minimum spanning tree (MST) was derived, which forms the backbone of the original network (40). Based on the MST, the betweenness centrality was estimated for each node to identify hubs. The betweenness centrality is defined as the number of shortest paths that pass through a node divided by the total number of shortest paths in the network (19). The broadband **betweenness centrality** for each ROI was used as the fourth metric.

For each of the four metrics, we additionally calculated six average measures per patient that incorporate information about the location and extent of the resection area: (1) the average of the ROIs overlapping with the resection (resection ROIs), (2) the average of all ROIs belonging to the lobe that contains the resection, (3) the average of the ROIs contralateral to the resection ROIs, (4) the average of all ROIs outside the resection cavity, (5) the difference between the average of the resection ROIs and the average of the contralateral ROIs, (6) the difference between the average of the resection ROIs and the average of the non-resection ROIs.

### Resection cavity

The resection cavity was determined for each patient from the 3-month post-operative magnetic resonance imaging (MRI) scan, which was normalized to the MRI template containing the AAL centroids. Subsequently the normalized post-operative MRI scan was linearly co-registered to the pre-operative MRI scan (used for MEG co-registration) using FSL FLIRT (version 4.1.6) with 12 parameter affine transformation. The resection cavity and ROI outlines were then used to visually determine the ROIs overlapping with the resection (resection ROIs).

### Group level analysis: group statistics

For each metric separately, we considered the ROIs with the five highest values and determined whether those ROIs overlapped with the resection area (i.e., corresponded to resection ROIs). We did that five times by choosing only the maximum value, the two highest values, and so on until the five highest values. Subsequently, we determined whether the number of patients with overlap was significantly higher than expected by chance using a binomial test. The probability of chance was estimated from a hypergeometric distribution (the discrete probability of drawing k out of m resection ROIs in n draws without replacement). For each of the thresholds of 1–5 ROIs, we calculated the probability of drawing at least one resection ROI (k ≥ 1) when randomly choosing 1–5 ROIs (*n* = 1, 2, 3, 4, or 5) from the 90 ROIs. For this calculation, we used the median number of resection ROIs (m = 7), as patients had a different number of resection ROIs (range 1–12). Additionally, we determined whether the number of patients with overlap differed between the seizure-free group and not seizure-free group using a Chi-square test of independence. Furthermore, we estimated whether the average measures differed between seizure-free patients and not seizure-free patients using an unpaired *t*-test. The analyses were performed in MATLAB (MATLAB and Statistics Toolbox Release 2012a, The MathWorks Inc., Natick MA, United States). All individual statistical tests were corrected for multiple comparisons using false-discovery rate (FDR) (41).

### Individual level analysis: machine learning

**Figure 1:**
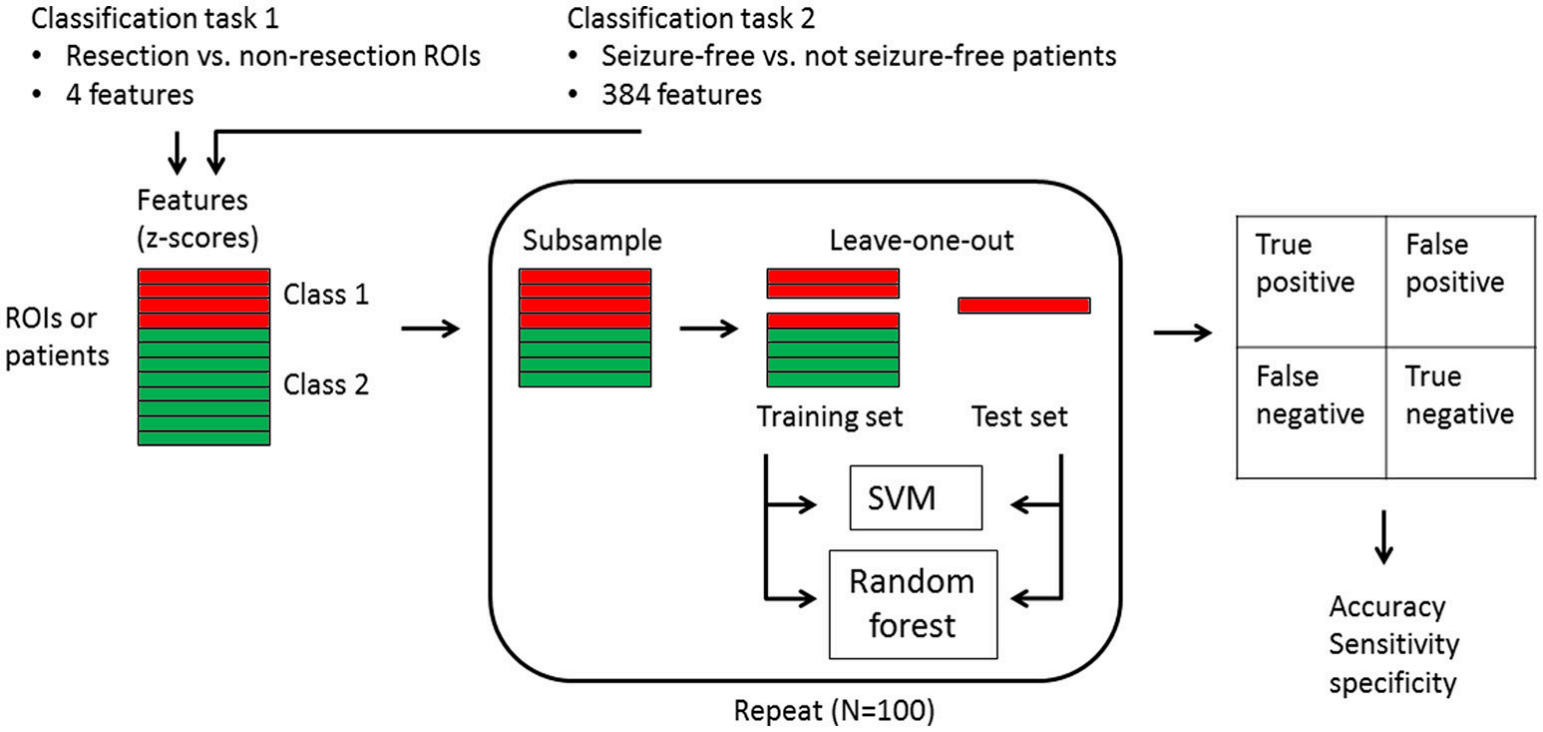
Flowchart of the machine learning classification. Two classification tasks were carried out: (1) 626 resection ROIs vs. 7834 non-resection ROIs in all patients with the four metrics for each ROI as input features, and (2) 64 seizure-free patients vs. 30 not seizure-free patients with the four metric values for all 90 ROIs plus the 24 averaged measures as input features. The features were first converted to z-scores, after which the majority class was subsampled 100 times. For each subsample round, one instance was kept apart as test set, while all other instances constituted the training set. Both a linear support vector machine (SVM) and a random forest were trained with the training set and predicted the class of the one test instance, which fell into one of the four categories of true positive, false positive, false negative, or true negative. The accuracy with confidence interval, as well as the sensitivity and specificity were derived from the results across the 100 test instances.

We used a linear support vector machine (SVM) and a random forest classifier. The SVM consisted of a linear kernel and was implemented from the LIBSVM library (42) (version 3.22, software available at http://www.csie.ntu.edu.tw/~cjlin/libsvm). The random forest was built from 500 trees, the number of features within a tree chosen at random for each decision split was set to the square root of the total number of features (MATLAB and Statistics Toolbox Release 2012a, The MathWorks Inc., Natick MA, USA). We applied the algorithms for two different classifications: resection vs. non-resected ROIs and seizure-free vs. not seizure-free patients (Figure 1). For the first classification, one class consisted of the resection ROIs in all 94 patients (626 resection ROIs) and the other class contained all other ROIs in all patients (7834 non-resection ROIs). The input for the classifiers was the four metrics for each ROI, yielding four features in total. For the second classification, one class contained the 64 seizure-free patients and the other class comprised the 30 patients with persistent seizures. The input per patient were the metric values for all 90 ROIs and the 24 averaged measures (six averaged measures for all four metrics), yielding 384 features in total. For both classifications, the features were converted to z-scores, using the mean and standard deviation across all ROIs or subjects for each feature. The class imbalance was corrected for by subsampling the majority class by randomly selecting 626 out of 7834 non-resection ROIs and 30 out of 64 seizure-free patients for the first and second classification task, respectively. The subsampling was repeated 100 times, and the accuracy, sensitivity, and specificity were averaged and the 95% confidence interval was calculated for the accuracy. The performance of the classifiers was tested with leave-one-out cross-validation.

## Results

### Group level

**Figure 2:**
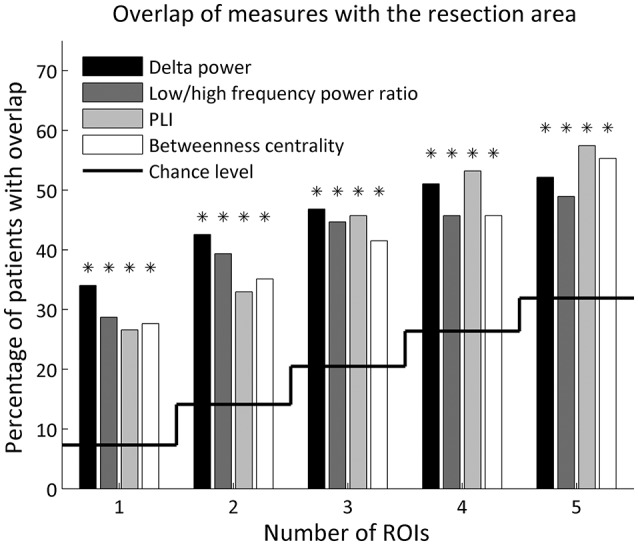
Percentage of patients with overlap of the resection area and the 1–5 ROIs with the highest values for each metric. Overlap by chance level is indicated by the solid line. A binomial test determined whether the number of patients with overlap was significantly above chance level (indicated with an asterisk), corrected for 20 tests using FDR.

The five ROIs with the highest values for all four metrics overlapped with the resection area (Figure 2 and Table 1). The overlap was highly significant compared to chance level, also after FDR correction. Considering only the ROI with the maximum value resulted in an overlap in about one-third of patients (28–34%, depending on the metric). When considering only a few ROIs, the delta power overlapped in the most patients (32 (34%), 40 (43%), 44 (47%) out of 94 patients when using 1, 2, 3 ROIs with the highest values, respectively), whereas when considering more ROIs, the PLI [50 (53%) and 54 (57%) patients when using 4 and 5 ROIs with the highest values, respectively] overlapped in the most patients, followed by betweenness centrality [52 (55%) patients for 5 ROIs].

**Table 1:** Number of patients (*n* = 94) in whom the metrics overlapped with the resection cavity for the 1–5 ROIs with the highest values.

	**1 ROI**			**2 ROIs**			**3 ROIs**			**4 ROIs**			**5 ROIs**		
**Metric**	**#Patients**	***p***	***p* corr**.	**#Patients**	***p***	***p* corr**.	**#Patients**	***p***	***p* corr**.	**#Patients**	***p***	***p* corr**.	**#Patients**	***p***	***p* corr**.
Delta power	32	< 0.001	< 0.001	40	< 0.001	< 0.001	44	< 0.001	< 0.001	48	< 0.001	< 0.001	49	< 0.001	< 0.001
Low/high frequency power ratio	27	< 0.001	< 0.001	37	< 0.001	< 0.001	42	< 0.001	< 0.001	43	< 0.001	< 0.001	46	0.003	0.003
PLI	25	< 0.001	< 0.001	31	< 0.001	< 0.001	43	< 0.001	< 0.001	50	< 0.001	< 0.001	54	< 0.001	< 0.001
Betweenness centrality	26	< 0.001	< 0.001	33	< 0.001	< 0.001	39	< 0.001	< 0.001	43	< 0.001	< 0.001	52	< 0.001	< 0.001

*All values were significantly larger than chance level (FDR corrected for 20 tests). P, uncorrected p-value; p corr, FDR-corrected p-value*.

The mean and standard deviation are given for each surgery outcome group and p-values of 24 unpaired t-tests after FDR correction. P, uncorrected p-value; p corr, FDR-corrected p-value.

**Figure 3:**
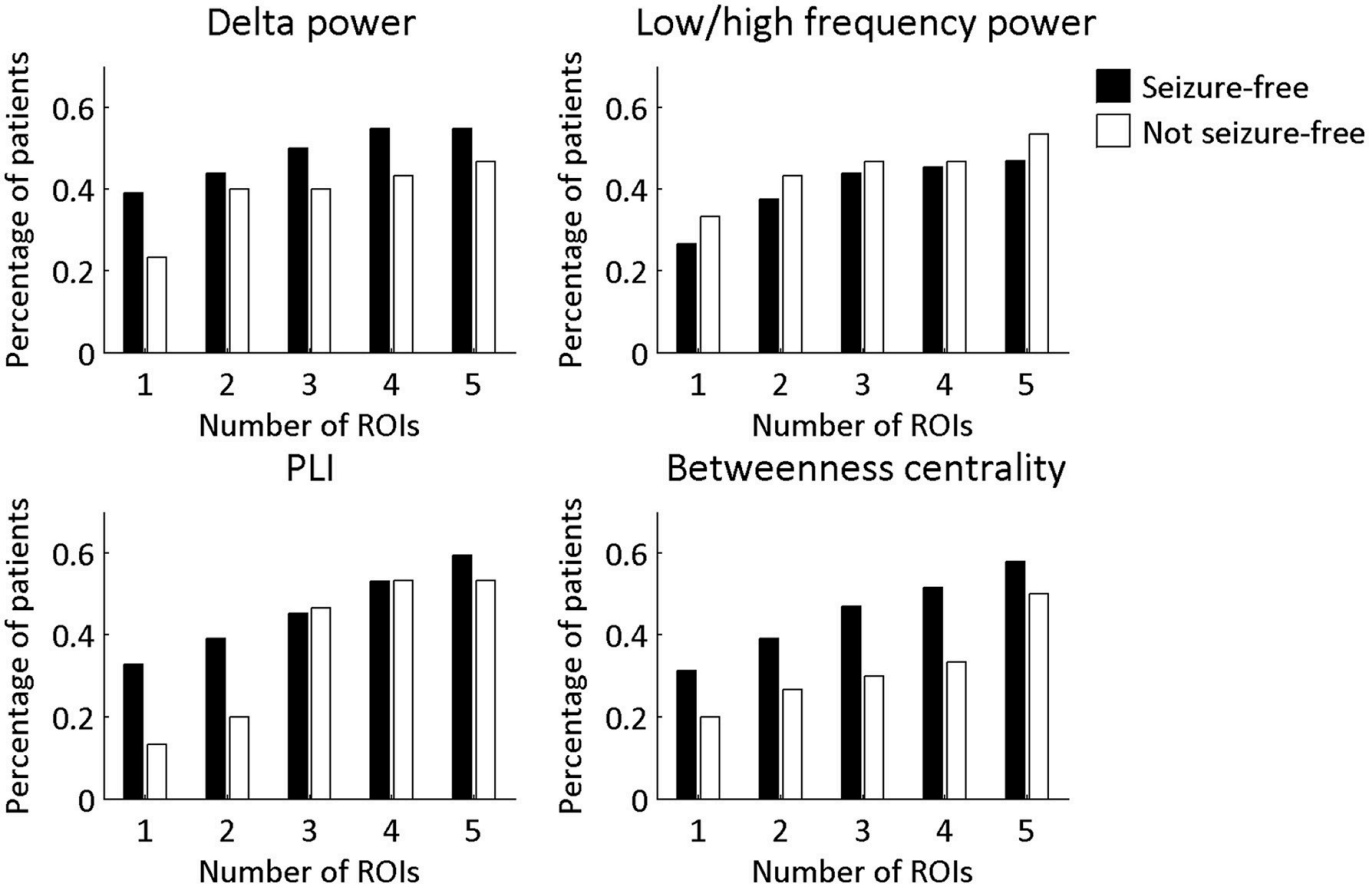
Percentage of patients in each surgery outcome group with overlap between the resection area and the four metrics. The ROIs with the 1–5 highest values were considered. A Chi-square test for independence was performed to determine if seizure-free patients and not seizure-free patients differed significantly in the percentage of patients with overlap. None of the differences remained significant after multiple comparison correction for 20 tests using FDR.

**Figure 4:**
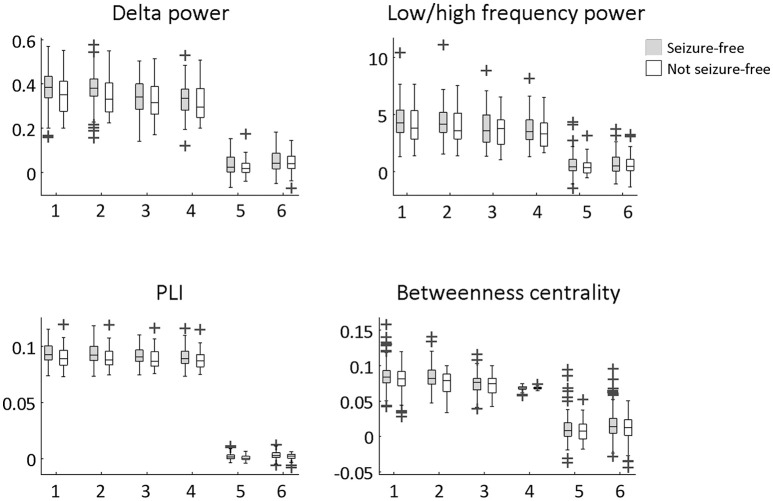
Difference between seizure-free and not seizure-free patients using the six averaged measures. The averaged measures were: (1) resection ROIs average, (2) resection lobe average, (3) contralateral resection ROIs average, (4) non-resection ROIs average, (5) difference between (1) and (3), (6) difference between (1) and (4). An unpaired t-test was performed to determine significant group differences. None of the differences remained significant after multiple comparison correction for 24 tests using FDR.

The overlap of the metrics with the resection area did not differ between seizure-free and not seizure-free patients (Figure 3 and Table 2). Before FDR correction, only PLI differed significantly between the two groups for the maximum ROI [$\chi_{\left(1\right)}^{2}$ = 3.97, *p* = 0.046]. Thus, even though the metrics overlapped with the resection area, this overlap did not discriminate between the two surgery outcome groups. The same was found for the averaged measures (Figure 4 and Table 3). Before FDR correction, only the average betweenness centrality was significantly higher in the resection lobe in the seizure-free patients compared to the not seizure-free patients [*t*_(92)_ = 2.41, *p* = 0.0179].

**Table 2:** Percentage of patients (64 SF and 30 NSF patients) in whom the metrics overlapped with the resection cavity for the 1–5 ROIs with the highest value.

	**1 ROI**				**2 ROIs**				**3 ROIs**				**4 ROIs**				**5 ROIs**			
**Metrics**	**SF(%)**	**NSF(%)**	***p***	***p* corr**.	**SF(%)**	**NSF(%)**	***p***	***p* corr**.	**SF(%)**	**NSF(%)**	***p***	***p* corr**.	**SF(%)**	**NSF(%)**	***p***	***p* corr**.	**SF(%)**	**NSF(%)**	***p***	***p* corr**.
Delta power	39	23	0.13	0.53	44	40	0.73	0.91	50	40	0.37	0.79	55	43	0.30	0.76	55	47	0.47	0.79
Low/high frequency power ratio	27	33	0.50	0.79	38	43	0.59	0.79	44	47	0.79	0.93	45	47	0.90	0.95	47	53	0.56	0.79
PLI	33	13	0.05	0.53	39	20	0.07	0.53	45	47	0.90	0.95	53	53	0.98	0.99	59	53	0.58	0.79
Betweenness centrality	31	20	0.26	0.73	39	27	0.24	0.73	47	30	0.12	0.53	52	33	0.10	0.53	58	50	0.48	0.79

*No group difference remained significant after FDR correction for 20 tests. SF, seizure-free; NSF, not seizure-free; p, uncorrected p-value; p corr, FDR-corrected p-value*.

**Table 3:** Difference between seizure-free and not seizure-free patients using averaged measures.

		**Seizure-free patients**		**Not seizure-free patients**			
		**Mean**	**std**	**Mean**	**std**	***p***	***p* corr**.
Delta power	Resection	0.3817	0.0910	0.3574	0.0851	0.2207	0.4264
	Resection lobe	0.3766	0.0854	0.3462	0.0827	0.1085	0.4264
	Contralateral	0.3449	0.0778	0.3304	0.0873	0.4208	0.4681
	Non-resection	0.3289	0.0747	0.3135	0.0779	0.3591	0.4681
	Difference to contralateral	0.0368	0.0495	0.0270	0.0455	0.3590	0.4681
	Difference to non-resection	0.0528	0.0485	0.0439	0.0463	0.4029	0.4681
Low/high frequency power ratio	Resection	4.4654	1.7443	4.0212	1.5541	0.2370	0.4264
	Resection lobe	4.3420	1.7207	3.9163	1.4334	0.2425	0.4264
	Contralateral	3.7916	1.5403	3.6020	1.4675	0.5738	0.5987
	Non-resection	3.7159	1.3100	3.3713	1.3468	0.2416	0.4264
	Difference to contralateral	0.6738	1.0309	0.4192	0.7953	0.2352	0.4264
	Difference to non-resection	0.7495	0.9682	0.6499	0.9653	0.6430	0.6430
PLI	Resection	0.0931	0.0091	0.0901	0.0100	0.1501	0.4264
	Resection lobe	0.0930	0.0090	0.0899	0.0099	0.1399	0.4264
	Contralateral	0.0910	0.0085	0.0892	0.0097	0.3546	0.4681
	Non-resection	0.0900	0.0082	0.0884	0.0089	0.3961	0.4681
	Difference to contralateral	0.0021	0.0031	0.0009	0.0023	0.0697	0.4264
	Difference to non-resection	0.0032	0.0038	0.0018	0.0032	0.0769	0.4264
Betweenness centrality	Resection	0.0867	0.0229	0.0784	0.0205	0.0933	0.4264
	Resection lobe	0.0844	0.0184	0.0748	0.0171	0.0179	0.4264
	Contralateral	0.0738	0.0155	0.0709	0.0150	0.3938	0.4681
	Non-resection	0.0680	0.0034	0.0686	0.0020	0.4291	0.4681
	Difference to contralateral	0.0130	0.0233	0.0075	0.0153	0.2487	0.4264
	Difference to non-resection	0.0187	0.0248	0.0099	0.0215	0.0956	0.4264

### Individual level

The four metrics were able to distinguish ROIs within the resection from ROIs outside the resection (Table 4). However, the effect was smaller at the individual level compared to the group level (compare with Table 1). The accuracy of the SVM was 59.94% (95% confidence interval: 59.67–60.22) and the accuracy of the random forest 60.34% (59.98–60.71).

**Table 4:** Classification of (1) resection vs. non-resection ROIs and (2) seizure-free vs. not seizure-free patients, using random forest and a linear support vector machine.

	**Accuracy**		**Sensitivity**	**Specificity**
	**Mean**	**Confidence interval**	**Mean**	**Mean**
**Resection vs. non-resection ROIs**				
Random forest	60.34%	59.98–60.71%	61.45%	59.52%
Support vector machine	59.94%	59.67–60.22%	55.07%	64.82%
**Seizure-free vs. not seizure-free patients (MEG metrics only)**				
Random forest	49.03%	47.25–50.82%	49.40%	48.67%
Support vector machine	43.77%	42.08–45.45%	42.60%	44.93%
**Seizure-free vs. not seizure-free patients (MEG and clinical metrics)**				
Random forest	49.74%	48.14–51.35%	50.55%	48.94%
Support vector machine	42.95%	41.54–44.36%	42.12%	43.77%

None of the classifiers could distinguish seizure-free patients from patients with persistent seizures using the four metrics for each ROI and the six averaged measures (Table 4). The SVM classifier gave an accuracy of 43.77% (95% confidence interval: 42.08–45.45) and the accuracy of the random forest was 49.03% (47.25–50.82). Adding clinical variables to the classifiers did not improve the differentiation between seizure-free and not seizure free patients (Supplementary Material).

## Discussion

The aim of this study was to identify metrics based on interictal MEG recordings that localize the epileptogenic zone. We found that all four evaluated metrics (delta power, low-to-high frequency power ratio, functional connectivity, and network hubs) localized the resection cavity in more patients compared to chance level. However, the localization of the resection cavity did not differ between seizure-free patients and patients with persistent seizures. At the individual level, we showed that machine learning classifiers could distinguish between resection areas and non-resection areas. However, similar to our findings on the group level, the classifiers could not distinguish between surgery outcome based on the four metrics.

### Localization of the resection area

For all four metrics, the highest values coincided with the resection area in more patients than expected at chance level. Relative delta power was the strongest indicator of the resection area, when the ROIs with the one to three highest values were considered. Focal slowing (i.e., delta activity) is known to indicate the epileptogenic zone in focal epilepsy (8, 9), perhaps on a lobar rather than sublobar level. It has been shown that increased delta activity localizes within or at the borders of the resection area in most patients (9) and lateralizes to the hemisphere containing the resection (8). Low-to-high frequency power ratio did not improve the localization ability above relative delta power alone.

Functional connectivity was increased in the resection area, which replicates earlier MEG and EEG findings (11, 14, 16). Englot and colleagues reported that patients with increased connectivity in the resection area were more likely to achieve seizure-freedom after surgery (43). Our result corroborates the hypothesis that the epileptogenic zone is functionally well connected within the brain network (13–15). Highly interconnected cells have been shown to exhibit enhanced network activity in a computational model of the rat dentate gyrus, resulting in a seizure-prone network (22). Similarly, increasing the interconnectedness by adding more long-distance connections to models of excitatory neurons in the hippocampus led network models to transition into seizure activity (44). This interconnectedness might lower the threshold for seizures (14).

The resection ROIs showed increased betweenness centrality, which points to the existence of hubs in the resection area. Elevated hub status in, or near, the epileptogenic zone has been reported by various studies (16–18, 21, 45). Similarly, the epileptogenic zone and seizure onset zone have been described as a driver, i.e., they exert strong influence over other brain regions (13, 46, 47). In addition, the removal of hubs has been associated with seizure freedom (18, 48). These findings suggest that the epileptogenic zone is a hub that influences other brain regions, and that it can be localized using connectivity and network measures.

The localization of the four metrics to the resection area was more pronounced at the group level compared to the individual level. The simplest explanation is that group differences can be found even when the differences are not significant in every patient. Dickten and co-workers, for example, reported that the seizure onset zone influenced other brain regions during the interictal state, but that this connection was only the strongest when averaging over the group (46). They reported that in more than one-third of the patients, the strongest connections were observed outside the seizure-onset zone. Most studies report group level findings, even though surgery is tailored to individual patients. It is therefore important to test localization strategies at an individual level, for example by using machine learning or individualized models (49).

### Resection area vs. epileptogenic zone

The evaluated metrics localized the resection area but not the epileptogenic zone. The metrics therefore achieved the same localization results as the presurgical evaluation by a multidisciplinary team of experts, but did not improve on this by predicting surgery outcome. To our knowledge, only one study so far predicted surgery outcome in a large and heterogeneous patient cohort regarding seizure etiology, which found that MEG spike location, when concordant with the resection area, predicted seizure freedom (2). Larger patient cohorts that are representative of the heterogeneous group of epilepsy surgery candidates, and with known surgery outcome, are needed to evaluate the many available metrics for epileptogenic zone localization.

### Surgery outcome

The four metrics that we evaluated did not differentiate between surgery outcome groups. The classification accuracy of the SVM was below 50%, which is probably because the learned patterns from the training dataset did not generalize to the test dataset. Nonetheless, other studies have found a relation to surgery outcome using similar metrics. For example, an MEG study has reported that the quantity of delta activity could be used as a predictor for surgery outcome (9), and Wilke et al. showed that the removal of regions with high betweenness centrality in invasive electrocorticography was associated with seizure freedom (18). In our study, both delta power and betweenness centrality showed a non-significant difference between outcome groups in the same direction as reported in the above studies. A possible explanation for the different results between studies is the difference in cohort size (94 vs. 25 patients), as spurious findings are more likely in small cohorts.

Alternatively, in our study the resection area may have been in the correct location for (at least part of) the patients with persistent seizures, but seizures persisted because the resection was not sufficiently extensive, did not fully remove tumor tissue in patients with tumor-related epilepsy, or tumor regrowth elicited new seizures. In the future, post-operative MEGs would be useful to detect the epileptiform or other abnormalities that might remain in patients who do not become seizure-free.

Several studies have proposed metrics that differentiate between seizure-free patients and patients with persistent seizures. Group differences were found with, for example, concordance of MEG dipole localization with the resection area (2), and MRI functional and structural connectivity analysis (50, 51). Other studies have successfully applied machine learning to predict surgery outcome (24–26). However, some predicting features were complex and difficult to interpret (25, 26), whereas features in our study were relatively straightforward and easily derived.

### Limitations

The five ROIs with the highest metric values were able to localize the resection area. However, considering five possible ROIs is not yet clinically relevant, as it remains unknown which one or more of the five ROIs to consider for surgery. Nonetheless, the location of each of the five ROIs can be compared to the findings of other presurgical evaluation modalities. Moreover, the ROI with the maximum value alone did also indicate the location of the resection area, which is clinically more applicable.

The division into surgery outcome groups was based on Engel classification 1 year after surgery, which is a common yet arbitrary time point. Longitudinal studies have shown that surgery outcome varies over time and that the number of patients achieving seizure freedom decreases over several years (52, 53). The estimation of surgery outcome at a different time point would likely have resulted in different groups and therefore possibly different results (54). Factors such as long-term tissue transformation or tumor growth can change surgery outcome at different time points (55). Exclusion of patients with tumors could result in a more reliable not-seizure free group, but for this study we wanted our patient cohort to be representative of all patients with epilepsy who undergo an MEG in our clinic. Future studies should investigate the differences between seizure-free and not seizure-free patients in homogeneous subgroups and at different time points after surgery.

We evaluated the highest values of four metrics based on results from previous studies. We could have analyzed the lowest values or extended those metrics with others, for example based on directed connectivity or other network measures. In addition to the MEG-based metrics we also included five clinical metrics in an extra analysis (see Supplementary Material), but this did not improve the classification of surgery outcome. In this study we concentrated on the most promising metrics for the time being. A next step could be to first develop metrics that differentiate between surgery outcome (50), and subsequently investigate whether such metrics also localize the epileptogenic zone.

## Conclusion

Localization of the epileptogenic zone is challenging in patients with a heterogeneous and complex etiology. We found that several metrics based on interictal MEG recordings localized the resection area but did not differentiate between seizure-free patients and patients with persistent seizures. The results demonstrate that metrics derived from interictal MEG recordings correspond to expert consensus derived from various presurgical evaluation modalities, but do not yet improve the localization of the epileptogenic zone. The next step is to develop metrics that localize the resection area in seizure-free but not in patients with persistent seizures. Machine learning is a useful tool to explore many such different metrics without the drawback of the multiple comparison problem. Those algorithms rely on a large quantity of data, which can only be provided by large patient cohorts or many segments of recorded data. Furthermore, machine learning tests hypotheses at an individual level, which is important in tailoring the surgical approach on a patient-by-patient basis in focal epilepsy irrespective of its etiology.

## Data availability

The datasets for this manuscript are not publicly available because the patients did not consent for the sharing of their clinically obtained data. Requests to access the datasets should be directed to Ida A. Nissen, i.nissen@vumc.nl.

## Author contributions

IN, CS, EvS, JR, and AH conceived the project. EvS, JB, PdW, SI, and DV acquired data. IN, CS, VW, and AH worked out the analysis methods. IN analyzed the data. IN, CS, EvS, VW, DV, and AH interpreted the results. IN, CS, EvS, VW, JR, JB, PdW, SI, DV, and AH wrote and edited the manuscript. All authors approved the submitted version.

### Conflict of interest statement

The authors declare that the research was conducted in the absence of any commercial or financial relationships that could be construed as a potential conflict of interest.
