# Supplementary material for: Localization of the Epileptogenic Zone Using Interictal MEG and Machine Learning in a Large Cohort of Drug-Resistant Epilepsy Patients
Source: Front Neurol. 2018 Aug 7;9:647. doi: 10.3389/fneur.2018.00647 (PMC6090046; doi:10.3389/fneur.2018.00647)
Supplement: Supplementary file 1 [file Table_1.docx]

Supplementary material

In addition to the MEG metrics, we included five clinical metrics: age at epilepsy onset (years), age at surgery (years), duration of epilepsy (years), gender (male or female), seizure type (simple seizures only, complex seizures and possibly simple seizures as well, generalized tonic-clonic seizures and possibly complex and simple seizures as well). Group differences for surgery outcome was tested with unpaired t-tests for the continuous clinical metrics and with chi-square tests for the categorical metrics. The clinical variables did not differ between surgery outcome groups after FDR correction (Table S1). For the machine learning classification between surgery outcome groups, missing values were imputed using the median of all patients. The classification did not improve when adding the clinical metrics to the MEG metrics (Table S2). This shows that for our cohort also the clinical metrics did not differ between seizure-free and not seizure-free patients on the individual level.

*Table S1: Difference between seizure-free and not seizure-free patients using clinical metrics. The median and interquartile range or number of patients are given for each surgery outcome group and p-values of 5 tests after FDR correction.*

|  | Data available | Seizure-free patients | | Not seizure-free patients | | | | |  |
| --- | --- | --- | --- | --- | --- | --- | --- | --- | --- |
| Continuous clinical metrics | | | | | | | | |  |
|  | # patients | median | interquartile range | median | interquartile range | *p* | | *p* corr. |  |
| Age at epilepsy onset | 81 | 11.79 | 5.08-18.50 | 20.50 | 9.02-31.98 | 0.0180 | | 0.0900 |  |
| Age at surgery | 92 | 35.46 | 22.69-48.23 | 44.79 | 32.79-56.79 | 0.0741 | | 0.1235 |  |
| Duration of epilepsy | 81 | 18.71 | 7.83-29.58 | 26.67 | 13.63-39.71 | 0.9663 | | 0.9663 |  |
| Categorical clinical metrics | | | | | | | | |  |
|  | # patients | # patients | | # patients | | *p* | | *p* corr. |  |
| Gender | 92 |  |  |  |  | 0.5004 | | 0.6255 |  |
| Male |  | 34 |  | 17 |  |  | |  |  |
| Female |  | 30 |  | 11 |  |  | |  |  |
| Seizure type | 65 |  |  |  |  | 0.0388 | | 0.0969 |  |
| Simple |  | 1 |  | 1 |  |  | |  |  |
| Complex |  | 30 |  | 5 |  |  | |  |  |
| Generalized  tonic-clonic |  | 15 |  | 11 |  |  |  | | |

*Abbreviations: p = uncorrected p-value; p corr. = FDR-corrected p-value*

Table S2: Classification of seizure-free versus not seizure-free patients with MEG and clinical metrics, using random forest and a linear support vector machine.

|  | Accuracy | | | Sensitivity | | Specificity | |
| --- | --- | --- | --- | --- | --- | --- | --- |
|  | Mean | Confidence interval | | Mean | | Mean | |
| Seizure-free vs. not seizure-free patients (MEG and clinical metrics) | | | | | | | |
| Random forest | 49.74% | | 48.14-51.35% | | 50.55% | | 48.94% |
| Support vector machine | 42.95% | | 41.54-44.36% | | 42.12% | | 43.77% |
